# In-vitro performance of a single-chambered total artificial heart in a Fontan circulation

**DOI:** 10.1007/s10047-021-01273-5

**Published:** 2021-05-06

**Authors:** Stephan Hildebrand, Sascha Groß-Hardt, Thomas Schmitz-Rode, Ulrich Steinseifer, Sebastian Victor Jansen

**Affiliations:** 1 Department of Cardiovascular Engineering, Institute of Applied Medical Engineering, RWTH Aachen University, Pauwelsstraße 20, 52074 Aachen, Germany; 2 Institute of Applied Medical Engineering, RWTH Aachen University, Aachen, Germany

**Keywords:** Fontan circulation, Mock circulation loop (MCL), Total artificial heart (TAH), Single ventricle, Congenital heart disease

## Abstract

An in-vitro study was conducted to investigate the general feasibility of using only one pumping chamber of the SynCardia total artificial heart (TAH) as a replacement of the single ventricle palliated by Fontan circulation. A mock circulation loop was used to mimic a Fontan circulation. The combination of both ventricle sizes (50 and 70 cc) and driver (Freedom Driver and Companion C2 Driver) was investigated. Two clinical relevant scenarios (early Fontan; late Fontan) as derived from literature data were set up in the mock loop. The impact of increased transpulmonary pressure gradient, low atrial pressure, and raised central venous pressure on cardiac output was studied. From a hemodynamic point, the single-chambered TAH performed sufficiently in the setting of the Fontan circulation. Increased transpulmonary pressure gradient, from ideal to pulmonary hypertension, decreased the blood flow in combinations by almost 2 L/min. In the early Fontan scenario, a cardiac output of 3–3.5 L/min was achieved using the 50 cc ventricle, driven by the Companion C2 Driver. Even under pulmonary hypertension, cardiac outputs greater than 4 L/min could be obtained with the 70 cc pump chamber in the late Fontan scenario. In the clinically relevant Fontan scenarios, implementation of the single chambered TAH performed successfully from a hemodynamic point of view. The replacement of the failing univentricular heart by a single chamber of the SynCardia TAH may provide an alternative to a complex biventricular repair procedure or ventricular support in Fontan patients.

**Supplementary Information:**

The online version contains supplementary material available at 10.1007/s10047-021-01273-5.

## Introduction

Establishment of the so-called Fontan circulation is a palliative surgical treatment for patients with an univentricular heart condition and is designed to overcome the absence of two separate ventricular chambers [1, 2]. Over time, many patients experience progressive failure of the Fontan circulation [2]. The causes of this failure include systolic and diastolic ventricular dysfunction as well as high pulmonary vascular resistance [3, 4]. Besides heart transplantation, which is still considered the gold standard, the focus shifts progressively towards the use of mechanical circulatory support due to a lack of donor organs [5–7]. Current clinical options for ventricular assist device (VAD) implantation in a Fontan patient without the take-down of the Fontan circulation are either cavopulmonary circulatory assistance with a right VAD or single ventricle assistance using a left VAD [8]. The implantation of a biventricular assist device (BiVAD) requires the take-down of the cavopulmonary anastomosis and the closure of a possible Fontan baffle fenestration, as described by Nathan et al*. *[9]. Beside several VADs, there is only one total artificial heart (TAH), the SynCardia TAH (SynCardia Systems LLC, Tucson, Arizona, USA) clinically available. The SynCardia TAH is composed of two individual pump chambers with dedicated compressed air connections that can be operated separately. Rossano et al*.* have reported the first successful use of the TAH as a bridge to transplantation for a Fontan patient in 2013. In this case, the authors claimed that the implantation of a TAH would restore a normal biventricular circulation. The patient recovered from multiple organ failure and received a heart transplantation on postoperative day 61 [10]. The TAH implantation requires the take-down (equal to BiVAD support) of the cavopulmonary junction and the creation of a neo atrium for the systemic venous return aggravating surgical complexity and risk [11]. Thus, such a bi-ventricular replacement of the single ventricle by a TAH is not feasible in many Fontan patients due to the vast variation in individual cardiovascular anatomy and unstable clinical condition.

The diverse situations leading to Fontan failure are complicating the discussion of the potential benefit of mechanical circulatory support. Valve insufficiency, ventricular thrombosis or arrhythmias may contraindicate the use of a VAD. Instead of supporting the ventricle with a VAD, these patients may benefit of the replacement of the single ventricle with a single-chambered TAH.

So far, the performance of a single-chambered SynCardia TAH as a replacement of the single ventricle in a failing Fontan circulation has not been investigated. In this case, the takedown of the anastomosis is not necessary, since only the failing ventricle is replaced.

In this study we investigated the in-vitro performance of a single-chambered SynCardia TAH in a mock Fontan circulation loop. Increased pulmonary vascular resistance and different levels of left atrial pressure were studied regarding their impact on the cardiac output of the single-chambered TAH. The aim of this study is to investigate the dependency of cardiac output (CO) in different load scenarios, defined by the transpulmonary pressure gradient.

## Materials and methods

A mock circulation loop (MCL) was used to simulate the peripheral pulmonary and systemic circulations to assess the performance of the single pump of the SynCardia TAH (SynCardia Systems LLC, Tucson, Arizona, USA) supporting the Fontan circulation [12]. The SynCardia 50 cc, with an approximate filling volume of 50 mL, generates a flow of up to 7.5 L/min, the 70 cc ventricle up to 10 L/min, respectively. The artificial ventricle was connected to a driver system via a pneumatic driveline. The two available driver systems, the Freedom Driver (FD, SynCardia Systems LLC, Tucson, Arizona, USA) and the Companion 2 Driver (CD, SynCardia Systems LLC, Tucson, Arizona, USA) generate a pneumatic and pulsatile pressure to the TAH through the drivelines.

### Mock Fontan circulation

**Fig. 1:**
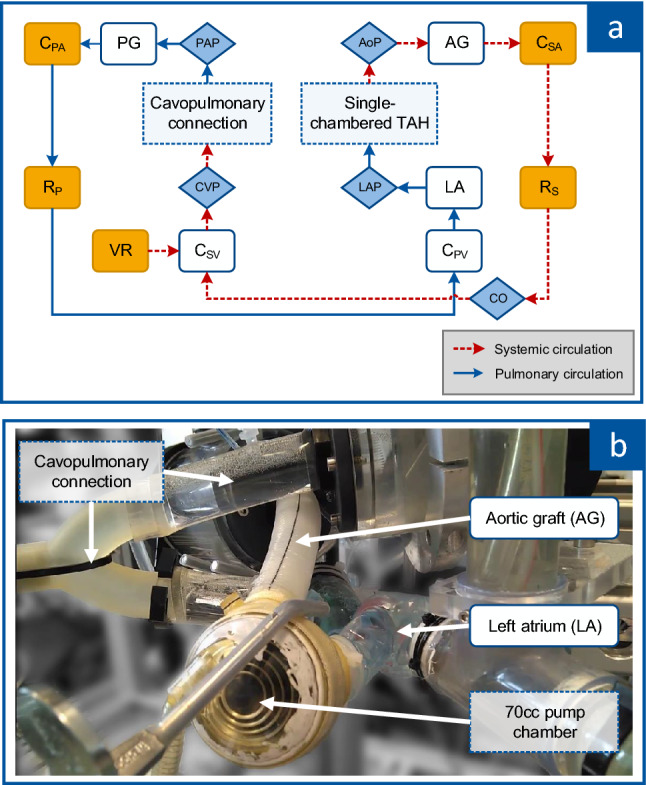
Mock circulation loop, **a** schematic: active elements (orange): systemic and pulmonary arterial compliance (*C*_SA_, *C*_PA_), systemic and pulmonary vascular resistance (*R*_S_, *R*_P_) and venous reservoir (VR); measurements (blue diamonds): aortic pressure (AoP), pulmonary artery pressure (PAP), left atrial pressure (LAP), central venous pressure (CVP), cardiac output (CO); left atrium (LA), pulmonary and aortic graft (PG, AG), systemic and pulmonary venous compliance (*C*_SV_, *C*_PV_); **b** picture of mounted 70 cc pump chamber as single-chambered TAH

The active MCL, described by Cuenca et al*.*, was adapted to mimic a Fontan circulation with an atriopulmonary connection [12]. This connection was created using a rigid tube connector with an inner diameter of 21 mm connecting the systemic venous circulation (right atrium) with the pulmonary arterial circulation (pulmonary graft adapter). The artificial ventricle was attached to the left atrium (LA) and connected to the arterial system by a SynCardia aortic graft. Figure 1 shows the schematic drawing of the complete MCL (a) and the single-chambered TAH (b).

The systemic and pulmonary circulations are imitated by active arterial compliance (*C*_SA_, *C*_PA_), active vascular resistance (*R*_S_, *R*_P_) and passive venous compliance (*C*_SV_, *C*_PV_). The filling volume, and, therefore, the mean filling pressure of the MCL, can be electrically varied by alternating the venous reservoir (VR).

The MCL was filled with an aqueous glycerol solution (40 mass-% glycerol) to mimic the viscosity of full blood with a normal hematocrit at 37 °C. A viscosity of 3.71 mPas at room temperature was achieved with a density of 1.106 g/cm^3^ [13].

The pressure and flow curves result from the performance of the single-chambered TAH in combination with the four-element Windkessel model (resistance, venous and arterial compliance, and fluid’s inertia) for the systemic and pulmonary circulation.

### Fontan hemodynamics

For a possible statement as to whether a single-chambered TAH is suitable for the failing Fontan circulation, two scenarios were considered. Gewillig et al*.* stated that *R*_P_ remains low for many decades, but is expected to increase at older age [14]. In contrast, many failing Fontan cases pertain to the pediatric population, hence the surgery is usually performed at the age of 1–5 years [15]. Therefore, a distinction has been made between the early Fontan patient (Table 1) and the late Fontan patient (Table 2).

**Table 1:** Patient specific data (age, body surface area) as well as hemodynamic values of cardiac output, pressures and pulmonary vascular resistance for the early Fontan scenario

	Authors		Experimental set point
	Yoshitake	Di Molfetta	
No. of patients	12	10 (30)^a^	Pump chamber size: 50 cc
BSA (m^2^)	0.5 ± 0.1	0.57 ± 0.31^a^	
Age (years)	2.5 ± 0.6	3.4 ± 3.7^a^	
CO (L/min)	2.3 ± 0.9	2.5 ± 0.9	–
CI (L/min/m^2^)	4.4 ± 1.3		–
MAP (mmHg)	63.4 ± 5.3	54.1 ± 18.1	60
PAP (mmHg)	11.8 ± 2.3	10.9 ± 4.2	–
LAP (mmHg)	7.3 ± 1.9	6.7 ± 2.9	–
RAP (mmHg)	–	–	8
*R*_P,Index_ (WU/m^2^)	1.8 ± 0.6	–	–
*R*_P_ (WU)	–	–	3.5
TPG (mmHg)	4.5^b^	4.2^b^	–

Values are represented as mean value ± standard deviation [15, 19]

*BSA* body surface area, *CO *cardiac output, *CI* cardiac index, *MAP* mean aortic pressure, *PAP* pulmonary artery pressure, *LAP, RAP* left and right atrial pressure, *R*_*P*_*, R*_*P,Index*_ pulmonary vascular resistance and pulmonary vascular resistance index, *TPG* transpulmonary pressure gradient

^a^Age and body surface refer to the whole patient cohort (*n* = 30); measurement data only to patients with Fontan procedure (*n* = 10)

^b^Calculated value

**Table 2:** Patient specific data (age, body surface area) as well as hemodynamic values of cardiac output, pressures and pulmonary vascular resistance for the late Fontan scenario

	Authors			Experimental set point
	Ohuchi	Schmitt	Mori	
No. of patients	23	10	18	Pump chamber size: 70 cc
BSA (m^2^)	–	1.4 ± 0.4	1.9 ± 0.29	
Age (years)	15.3 ± 8.0	22 ± 10	29.2 ± 7.3	
CO (L/min)	–	2.97 ± 0.76	–	–
CI (L/min/m^2^)	2.6 ± 0.6	2.2 ± 0.7	2.8 ± 0.9	–
MAP (mmHg)	70 ± 8	–	83.5 ± 14.3	80
PAP (mmHg)	–	11.1 ± 1.3	–	–
LAP (mmHg)	–	–	13.5 ± 5.7	–
RAP (mmHg)	11.4 ± 2.2	–	18.6 ± 6.5	15
*R*_P,Index_ (WU/m^2^)	1.8 ± 0.6	2.7 ± 1.0		–
*R*_P_ (WU)	–	3.7 ± 2.0	–	3.5
TPG (mmHg)	–	5.5 ± 1.3	5.1 ± 3.9	–

Values are represented as mean value ± standard deviation [16–18]

*BSA* body surface area, *CO* cardiac output, *CI* cardiac index, *MAP* mean aortic pressure, *PAP* pulmonary artery pressure, *LAP, RAP* left and right atrial pressure, *R*_*P*_*, R*_*P,Index*_ pulmonary vascular resistance and pulmonary vascular resistance index, *TPG* transpulmonary pressure gradient

Echocardiographic and hemodynamic data of Fontan patients from different published studies, are shown as mean value ± standard deviation [15–20]. In younger Fontan patients (age < 5 years) a CO of 2.3–2.5 L/min with a mean atrial pressure of 6.7–7.3 mmHg was measured, while the pulmonary artery pressure (PAP) and the transpulmonary pressure gradient (TPG) remained low [15, 19, 20]. In the later stage of the Fontan circulation the mean CO increased above 3 L/min with an atrial pressure of 13.5 mmHg and the mean aortic pressure ranging from 79 to 83.5 mmHg [16–18]. Whenever possible, we calculated the values for the transpulmonary pressure gradient. Pulmonary hypertension is considered for TPG values of 12 mmHg or higher [21]. Patients with a TPG less than 8 mmHg had greater freedom from palliation failure [22]. This value is utilized as an upper limit of the ideal range for the TPG.

### Experimental design and parameter variation

The parameters for *C*_SA_ and *C*_PA_ in the MCL were set to 1.1 mL/mmHg and 3.1 mL/mmHg, respectively, representing normal cardiovascular conditions for the systemic and pulmonary compliances. *R*_S_ was used to achieve the aortic pressure of the experimental set point. It was kept constant during the series of experiment, characterizing a physiological vascular resistance [12, 23].

Due to the cavopulmonary circulation, the pulmonary artery pressure (PAP) is mainly defined by the central venous pressure (CVP). By changing the volume of the VR, the filling pressure of the simulator can be adjusted. In this way, the target value of the CVP is matched to the experimental set point.

The *R*_P_ was increased starting with a value of $0.08{\text{ }}\frac{{{\text{mmHg s}}}}{{{\text{mL}}}}$ (= 13 Woods Unit), which corresponds to a healthy pulmonary vascular resistance. Different levels of pulmonary hypertension were simulated with values ranging from $0.2{\text{ to }}0.5{\text{ }}\frac{{{\text{mmHg s}}}}{{{\text{mL}}}}$ (= 3.3–8.1 Woods Unit) [21].

As a measure of pulmonary hypertension, the TPG was calculated as explained below and used in the result section. The transpulmonary pressure gradient is defined as the difference of mean pulmonary artery and left atrial pressure (LAP), which is directly influenced by changes in pulmonary resistance:

1 $$\mathrm{TPG}=\mathrm{PAP}-\mathrm{LAP}=\mathrm{CO}\bullet {R}_{\mathrm{P}}$$

To compare the results of the different drivers, the driver pressure (205 mmHg), the driver vacuum (− 10 mmHg) and the systole duration (50%) of the CD were set to the predefined values of the FD [24].

For each combination of test conditions, the TAH systems were subjected to a range of heart rates (HR) between 100 and 150 min^−1^. Please note, that this exceeds the normal operating range of the FD, which is specified to be 120 ± 15 min^−1^ [24].

Three scenarios will be considered in the hemodynamic study:

1. Influence of TPG on the pump flow.
2. Influence of CVP on the pump flow.
3. Influence of LAP, representing the preload to the ventricle as a result of scenario 1 and 2.

### Data recording and analysis

Calibrated flow sensors (20PXL; TS420 Flow Module, Transonic Systems, Ithaca, NY, USA) measured the CO of the single-chambered TAH. Inlet and outlet pressures of the TAH and the Fontan connection [aortic pressure (AoP), PAP, LAP and CVP] were measured by pressure transducers (DPT-9009; CODAN, Lensahn, Germany), using an analog amplifier (GSV-1A8, ME-Messsysteme, Henningsdorf, Germany). All analog signals were digitalized with a sample rate of 1 kHz using the HIL-System dSPACE DS1103 (dSPACE, Paderborn, Germany). For each set of parameters, the measurement was recorded for 120 s once a steady state of parameters was reached. Arithmetic mean values were calculated in post-processing.

## Results

The experimental set points for the early and late Fontan scenario were achieved with the single-chambered TAH under normal pulmonary resistance. A figure with plotted flow and pressure curves of the set points can be found in the electronic supplementary material (experimental set point A and B).

The figure shows a 3-s data-record for the representative combination of the 50 cc pump chamber (early Fontan) and the 70 cc pump chamber (late Fontan) driven by the CD with a HR of 100 min^−1^.

### Influence of transpulmonary pressure gradient

**Fig. 2:**
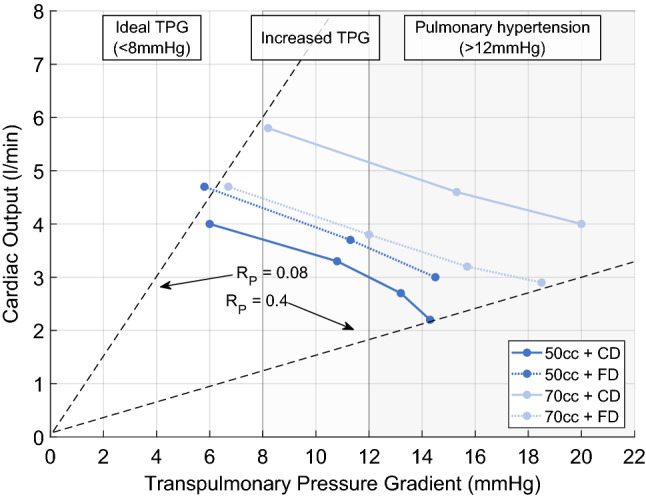
Cardiac output depending on transpulmonary pressure gradient (TPG); with increase of pressure gradient cardiac output decreases for all four combinations. Pulmonary hypertension is considered for TPG values of 12 mmHg or higher [21]. Patients with a TPG less than 8 mmHg had greater freedom from palliation failure [22]. This value is utilized as an upper limit of the ideal range

Over a wide range of TPG, all four combinations delivered a CO above 2 L/min. An increase in pressure gradient decreased the CO. Figure 2 depicts the course of the CO as a function of TPG as well as the ranges for an ideal TPG [22] and the definition of pulmonary hypertension [21]. The larger pump chamber driven by the CD delivered between 5.8 and 4 L/min for a TPG of 8.2 and 20 mmHg, respectively.

### Influence of central venous pressure

**Fig. 3:**
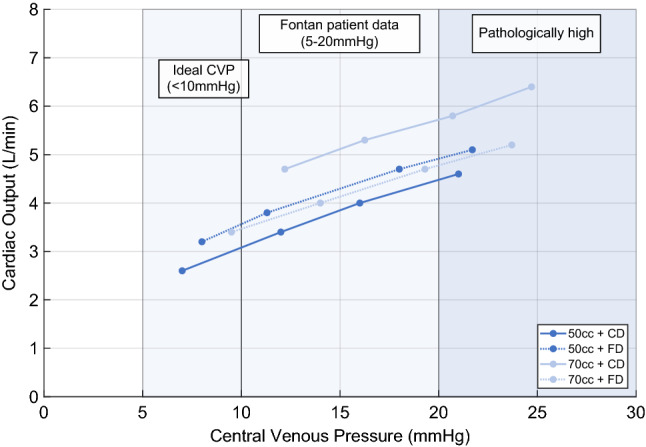
Cardiac output as function of central venous pressure (CVP) and three representing areas; (1) “Optimal” CVP lies below 10 mmHg for late survivors (> 15 year follow-up) [29]; (2) Masutani et al*.* measured a CVP of 5–20 mmHg on Fontan patients [30]; (3) CVP greater than 20 mmHg is considered pathologically high

As the VR is lowered, the filling volume of the hydraulic simulator, and therefore, the filling pressure are increased. The mean filling pressure of the MCL represents the CVP in a patient. Figure 3 shows the dependency of CO on CVP for a constant *R*_P_ value of $0.08\;\frac{{{\text{mmHg s}}}}{{{\text{mL}}}}$, corresponding to a normal pulmonary vascular resistance, and for the combinations of pump chamber size and the driver choice. CO is calculated as the mean value over the entire BPM range.

A higher CVP results in a higher CO independent of driver choice or pump chamber size. The FD shows no difference of CO dependent on pump chamber size. The highest CO was archived with the 70 cc pump chamber powered by the CD.

### Influence of left atrial pressure

**Fig. 4:**
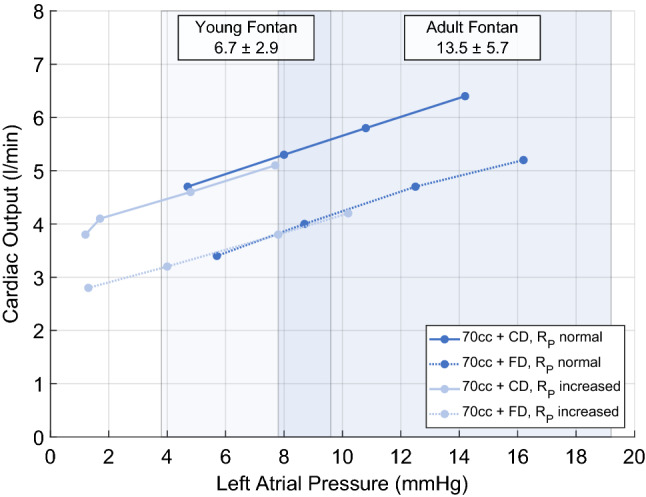
Cardiac output as a function of left atrial pressure (LAP) for a normal pulmonary vascular resistance (*R*_P_) and a raised *R*_P_, simulating pulmonary hypertension. The graphs show the combination of the Freedom Driver (FD) and Companion C2 Driver (CD) with the 70 cc ventricle. In young Fontan patients, the LAP is 3.8 and 9.6 mmHg [15], in adult patients between 7.8 and 19.2 mmHg [18]. The higher the preload of the ventricle the higher the cardiac output, independent of the *R*_P_

In addition to the TPG, a preload sensitivity of the SynCardia’s pump chamber was observed. LAP directly affected the preload of the single ventricle. The Companion 2 Driver in combination with the 70 cc pump chamber provided a flow of 4.7 ± 0.1 L/min for LAP of 4.7 ± 0.5 mmHg, respectively, 6.4 ± 0.3 L/min for a LAP of 14.2 ± 1.0 mmHg over the full range of BPM and a normal *R*_P_. For an increase of pulmonary vascular resistance, a decreased LAP was observed. Figure 4 illustrates the sensitivity of CO on LAP for the 70 cc pump chamber in combination with both, a normal and a raised *R*_P_. Hemodynamic data from young patients (age 2.5 ± 0.6 years) [15] and adult patients (29.2 ± 7.3 years) are shown for reference [18].

## Discussion

This study is the first to characterize the suitability of a single TAH ventricle in a Fontan circulation as a possible single-chambered replacement and describes the underlying limitation for the CO. The aim was to investigate the influence of CVP, TPG and LAP on CO.

When the CVP was increased, all four combination showed a raised CO in the experiment. Although the CVP can be chronically elevated in Fontan patients, a high CVP may lead to vascular congestion, edema, ascites or lymphatic failure [25]. Raised venous pressure may involve diverse secondary complications, but lead to an increased CO of the single-chambered TAH.

With an increase of LAP the CO rises, the TAH pump chamber demonstrates a Frank-Starling-like behavior. Since this behavior is not limited by the Fontan circulation, these findings agree with the physiological characterization of the SynCardia TAH under biventricular use and with clinical observation of patient’s hemodynamics [26, 27].

With reference to the classification of VAD support in Fontan patients by Buratto et al. the application of a single-chambered TAH can be discussed for both scenarios [28]. Patients with a systolic ventricular dysfunction develop a raised venous pressure. As we could show in this study, this has a favorable effect on the CO of the TAH (see Fig. 3). A moderate or severe pulmonary hypertension is limiting the preload to the ventricle and decreases the CO. A statement about the applicability, therefore, depends strongly on the patient’s individual cardiac demand and needs to be addressed on a patient-specific basis. In the second scenario, patients with a preserved ventricular function may benefit from a sub-pulmonary VAD support. This support is not feasible with a single-chambered TAH as a replacement of the failing single ventricle. In the later stages of the disease, patients may develop a ventricular dysfunction [14, 25]. In this scenario the presented use of the single-chambered TAH may be beneficial.

### Early Fontan patient scenario

For the early Fontan patient the 50 cc pump chamber was chosen due to its reduced dimensions. In combination with the CD, the 50 cc generated a MAP of 66–75 mmHg over the full range of HR, while the LAP lay between 5 and 7 mmHg. With the increased *R*_P_ a TPG of 10 mmHg was created, the PAP remained constant at about 17 mmHg. In these setting points a CO with a value of 3–3.5 L/min was measured. Based on these values the assumption may be derived that the 50 cc could be used as a single-chambered replacement for a possible bridge to transplantation in early Fontan patient.

### Late Fontan patient scenario

The larger 70 cc pump chamber created an AoP of 80–87 mmHg, for the complete range of HR, driven by the FD. The atrial pressure amounted to about 7 mmHg. The *R*_P_, with a value of 0.3 mmHg s/mL, created an increased TPG of 16–18 mmHg. This value is considered as a severe pulmonary hypertension. Although the CO lay with 3.4–3.8 L/min in the range of the target value, a high PAP and CVP of 24 mmHg have resulted. These pressure most likely will cause secondary complications. A definite statement about the application in the late Fontan scenario is, therefore, not or only partially possible.

### Limitations and future research

There are limitations regarding the in vitro test method used in this study and the presented results. The study investigated the SynCardia TAH system as it is the only clinically available TAH. The cavopulmonary connection was created using a rigid tubing with a defined diameter. The geometry, shape, and dimensions of the cavopulmonary connection directly influence its flow resistance. Beside this connection a possible fenestration in the Fontan prosthesis impacts the hemodynamics as it bypasses the pulmonary circuit. The age of the patient as well as the creation of the Fontan circulation have an influence on the value of systemic and pulmonary compliances. In the experimental study both values were kept constant, representing physiological values. In the study design the driver pressure and driver vacuum pressure were kept constant to enable the comparison between both driver systems. An increase in the vacuum driver pressure may result in a better filling of the pump chamber, hence a higher CO. This in vitro study was limited to the hemodynamic behavior of the single-chambered TAH. Further important effects, such as the impact of the diminished pulsatility and the end organ perfusion could not be addressed. A low CO paired with an increased CVP may lead to protein losing enteropathy as a secondary complication of the failing Fontan circulation.

## Conclusion

The application of a single-chambered TAH as a ventricular replacement in the Fontan circulation showed promising results in the MCL. The TPG was found to be the main limiting factor of CO. For the early Fontan patient scenario, the 50 cc ventricle met the target values for the required blood flow while generating a sufficient aortic pressure even in the presence of pulmonary hypertension. In the late Fontan scenario, these properties could only be partially confirmed due to a high PAP and CVP, most likely causing secondary complications, while a sufficient CO for the complete range of TPG was achieved.

These results suggest that, compared to hemodynamic patient data from the literature, it is technically possible to maintain a physiological range of blood flow with a single artificial ventricle as a single-chambered TAH palliated with the Fontan circulation. Therefore, this may be an option as mechanical circulatory support without the need of Fontan take-down for patients which are not suitable for biventricular replacement nor ventricular support.

## Supplementary Information

Below is the link to the electronic supplementary material.

Supplementary file1 Aortic pressure (AoP), cardiac output (CO), left atrial pressure (LAP) and pulmonary artery pressure (PAP) represent the experimental set point A, (early Fontan scenario) measured with 50 cc pump chamber driven by the Companion C2 Driver (CD) with a heart rate of 100 min^−1^ (PDF 183 KB)

Supplementary file2 Aortic pressure (AoP), cardiac output (CO), left atrial pressure (LAP) and pulmonary artery pressure (PAP) represent the experimental set point B, (late Fontan scenario) measured with 70 cc pump chamber driven by the Companion C2 Driver (CD) with a heart rate of 100 min^−1^ (PDF 182 KB)
