# Supplementary figures and images for: In-vitro performance of a single-chambered total artificial heart in a Fontan circulation
Source: J Artif Organs. 2021 May 6;25(1):1–8. doi: 10.1007/s10047-021-01273-5 (PMC8866354; doi:10.1007/s10047-021-01273-5)

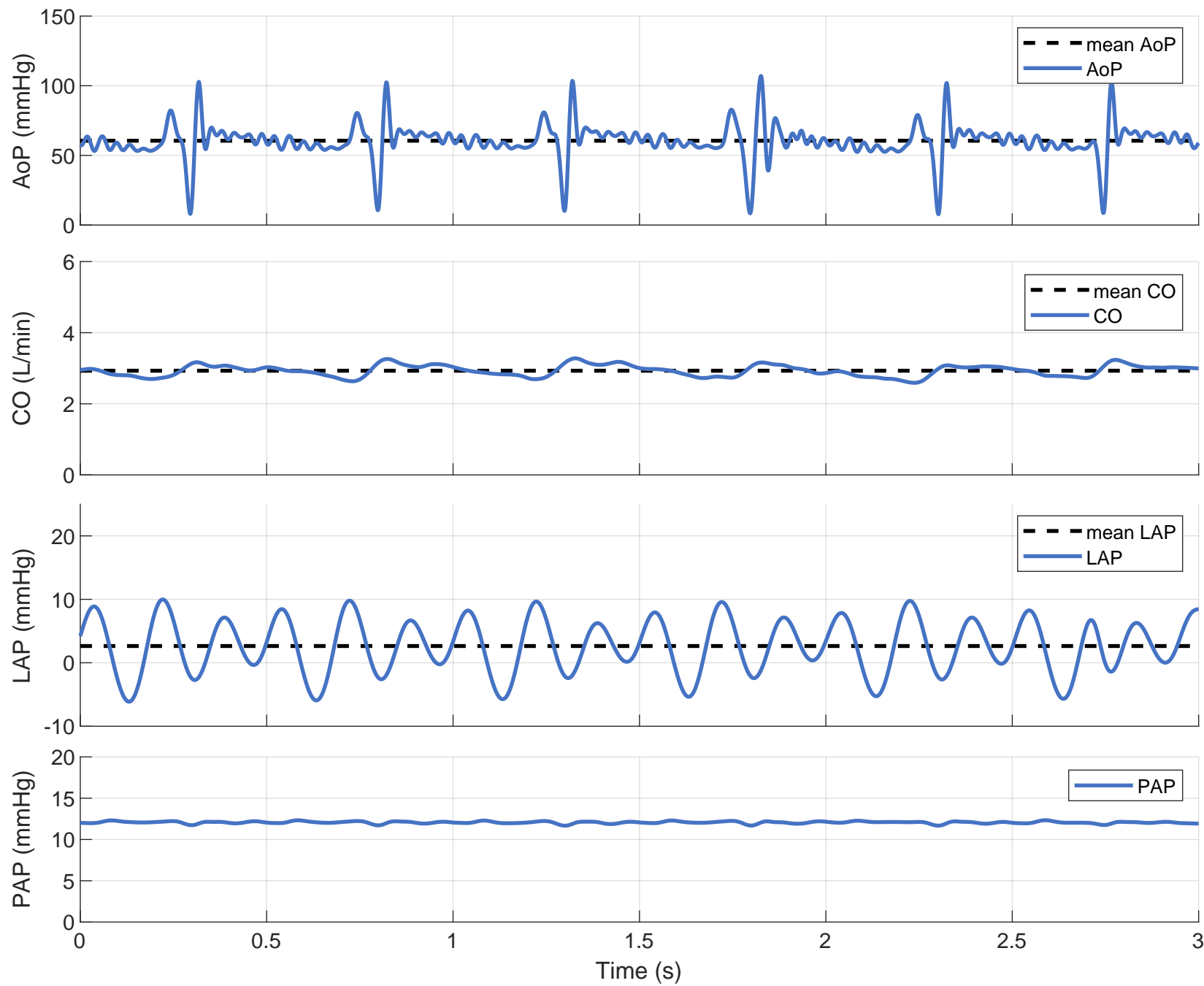

Supplement: Supplementary file 1 — Supplementary file1 Aortic pressure (AoP), cardiac output (CO), left atrial pressure (LAP) and pulmonary artery pressure (PAP) represent the experimental set point A, (early Fontan scenario) measured with 50 cc pump chamber driven by the Companion C2 Driver (CD) with a heart rate of 100 min−1 (PDF 183 KB) [file 10047_2021_1273_MOESM1_ESM.pdf]

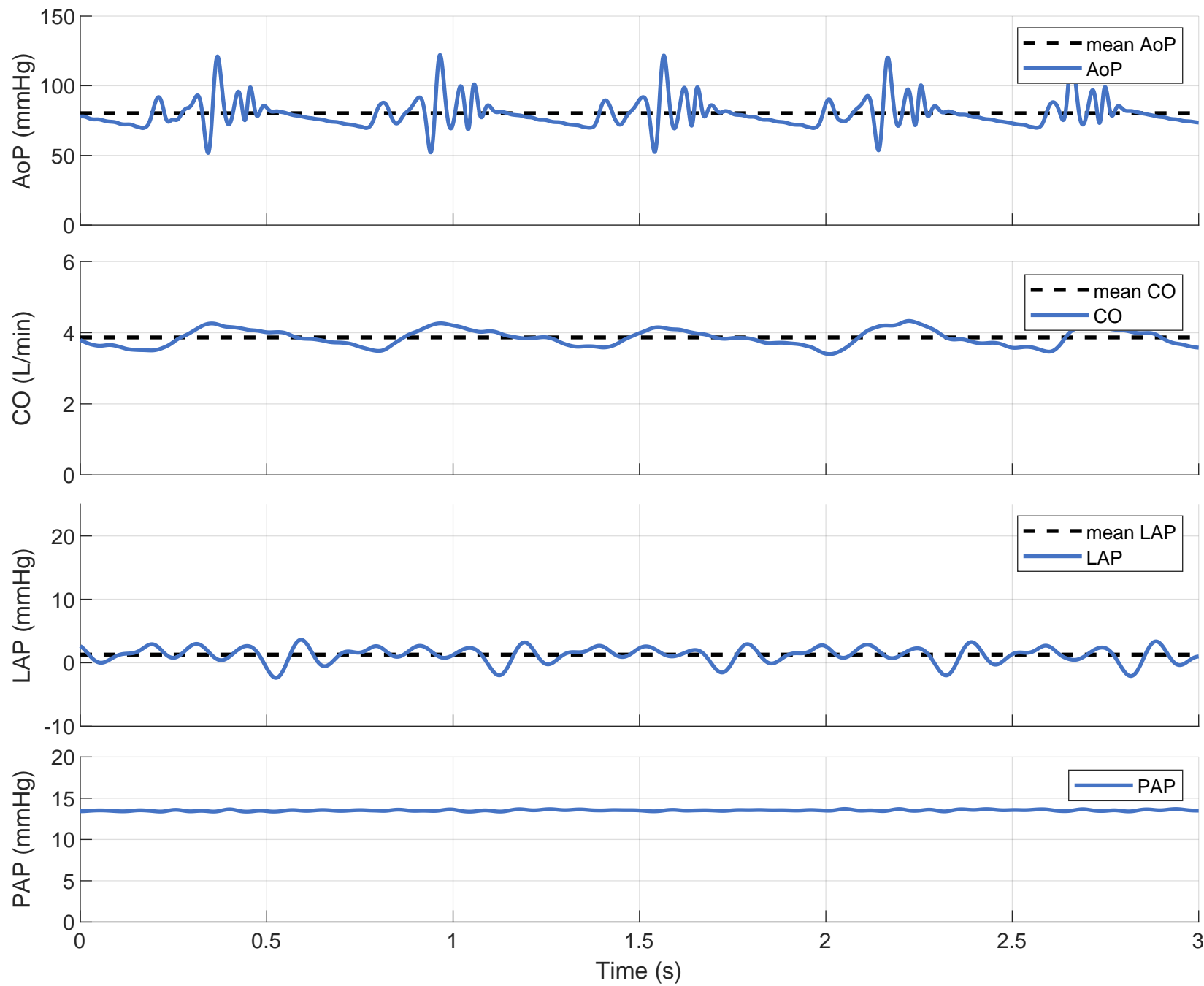

Supplement: Supplementary file 2 — Supplementary file2 Aortic pressure (AoP), cardiac output (CO), left atrial pressure (LAP) and pulmonary artery pressure (PAP) represent the experimental set point B, (late Fontan scenario) measured with 70 cc pump chamber driven by the Companion C2 Driver (CD) with a heart rate of 100 min−1 (PDF 182 KB) [file 10047_2021_1273_MOESM2_ESM.pdf]
